# ScanNeo2: a comprehensive workflow for neoantigen detection and immunogenicity prediction from diverse genomic and transcriptomic alterations

**DOI:** 10.1093/bioinformatics/btad659

**Published:** 2023-10-26

**Authors:** Richard A Schäfer, Qingxiang Guo, Rendong Yang

**Affiliations:** Department of Urology, Northwestern University Feinberg School of Medicine, Chicago, IL 60611, United States; Department of Urology, Northwestern University Feinberg School of Medicine, Chicago, IL 60611, United States; Department of Urology, Northwestern University Feinberg School of Medicine, Chicago, IL 60611, United States; Robert H. Lurie Comprehensive Cancer Center, Northwestern University Feinberg School of Medicine, Chicago, IL 60611, United States

## Abstract

**Motivation:**

Neoantigens, tumor-specific protein fragments, are invaluable in cancer immunotherapy due to their ability to serve as targets for the immune system. Computational prediction of these neoantigens from sequencing data often requires multiple algorithms and sophisticated workflows, which are currently restricted to specific types of variants, such as single-nucleotide variants or insertions/deletions. Nevertheless, other sources of neoantigens are often overlooked.

**Results:**

We introduce ScanNeo2 an improved and fully automated bioinformatics pipeline designed for high-throughput neoantigen prediction from raw sequencing data. Unlike its predecessor, ScanNeo2 integrates multiple sources of somatic variants, including canonical- and exitron-splicing, gene fusion events, and various somatic variants. Our benchmark results demonstrate that ScanNeo2 accurately identifies neoantigens, providing a comprehensive and more efficient solution for neoantigen prediction.

**Availability and implementation:**

ScanNeo2 is freely available at https://github.com/ylab-hi/ScanNeo2/ and is accompanied by instruction and application data.

## 1 Introduction

Neoantigens are foreign protein fragments that originate from genetic mutations within cancer cells and are entirely absent in normal tissue. When presented on major histocompatibility complex (MHC) molecules, these neoepitopes can be recognized by CD4${}^{+}$ or CD8${}^{+}$ T-cells. This can trigger an anti-cancer immune response in patients. However, cancer cells have developed resistance to anti-cancer immunity (Zhu *et al.* 2021). This effect can be reversed by cancer immunotherapies, which e.g. improve the presentation of neoepitopes. For that, tumor-specific neoantigens need to be identified to improve adoptive T-cell therapies. In the past, ScanNeo (Wang *et al.* 2019) has been developed for detecting insertion and deletion (indel)-derived neoantigens and later combined with ScanExitron (Wang *et al.* 2021) to detect neoantigens from exitron-derived events (Wang and Yang 2021). While several tools have been created to identify neoantigens from sequencing data, most focus mainly on finding neoantigens that come from single-nucleotide variants (SNVs), or indel events. But, they often overlook other sources of neoantigens like gene fusions or alternative splicing. In that regard, pVACtools (Hundal *et al.* 2020) provides a toolkit for neoantigen prediction and visualization, but requires a list of identified non-synonymous somatic variations identified by other pipelines. However, this requires the integration of multiple tools that need to be coordinated with one another. This can be simplified using workflow management systems, such as the Nextflow workflow language (Di Tommaso *et al.* 2017) or the snakemake workflow management system (Mölder *et al.* 2021). For instance, nextNEOpi (Rieder *et al.* 2022) presents a comprehensive workflow implemented in Nextflow but uses most routines from pVACtools. Features of existing workflows for the prediction of neoantigens are listed in Supplementary Table S1. To better address the complexity of coordinating tools and overcome these limitations, we present ScanNeo2. This advanced snakemake workflow is designed to discover neoantigens from a broader array of sources, offering a holistic and streamlined method to predict neoantigens from sequencing data.

## 2 Results

### 2.1 Neoantigen detection from multiple sources

**Figure 1.**
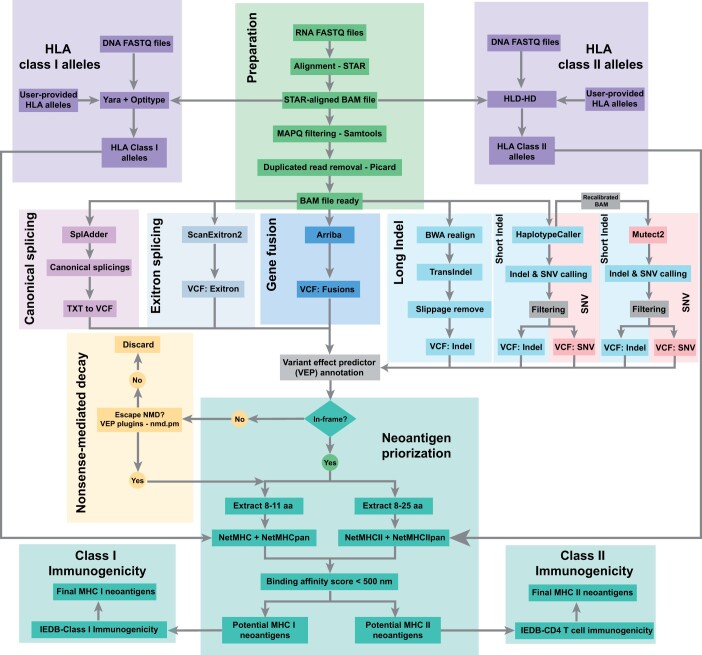
The workflow of ScanNeo2 is divided into three parts. In the first part, the input reads are prepared for the subsequent analysis, which includes the genotyping of the HLA alleles. In the next step, the variation calling is performed using the different modules canonical splicing, exitron, gene fusion, and indel/snp detection. This is concluded with the neoantigen prioritization.

To date, various methods have been established for detecting genomic mutations and transcriptomic variants. We took advantage of those tools and developed a snakemake-based workflow for the detection of neoantigens from multiple sources, termed ScanNeo2 (Fig. 1). It takes as input both raw and processed WGS/WES data and/or RNA-seq data from normal/tumor samples. In addition, it also accepts a list of already identified variants in “Variant Call Format.” In principle, ScanNeo2 consists of different steps described in the following. Based on the provided data, the sequencing reads can be pre-processed, which includes quality filtering or removal of adapter sequences. For that, fastp (Chen *et al.* 2018) is applied, and the reads are then subjected to the genotyping of HLA alleles and the mutation calling. In the former, DNA-seq and RNA-seq data are filtered for HLA reads using yara (Siragusa *et al.* 2013) and subsequently aligned against a panel of HLA class I alleles using Optitype (Szolek *et al.* 2014). This results in a list of detected 4-digit HLA genotypes that are later used in the prediction MHC class I antigen presentation. In a similar manner, HLA-HD (Kawaguchi *et al.* 2017) is used for HLA class II genotyping. In the following, the RNA-seq data are aligned against the reference genome using BWA (Li and Durbin 2009), and subsequently filtered for mapping quality, duplicates, and discarded of unmapped reads. In the mutation calling, the processed reads are subjected to SplAdder (Kahles *et al.* 2016) for the detection of alternative splicing events, and ScanExitron for identifying exitron-splicing events. In addition, gatk (Van der Auwera and O’Connor 2020) is utilized for detection of short indels, as well as SNVs. Here, the modules mutect2, and haplotypecaller are used for identifying somatic and germline variants, respectively. This is combined with transIndel (Yang *et al.* 2018) to detect long variants. ScanNeo2 also incorporates gene fusion events. For that, the aligned reads are first realigned using STAR (Dobin *et al.* 2013), and then subjected to Arriba (Dobin *et al.* 2013). For the individual outputs of the respective tools, the (VCF) is used to determine the downstream effects of the variants. In the case of a frameshift, only the transcripts are further considered that escape nonsense-mediated decay. The next step is to extract the amino acid sequence of the transcripts surrounding the variant. For that, peptides of different lengths are extracted that span the variants. This include peptides of length 8–11 and 13–25 amino acids, corresponding to MHC class I and MHC class II peptides, respectively. In the following, the Immune Epitope Database and Analysis Resource (IEDB) (Dhanda *et al.* 2019) is used to predict the T-cell epitope for MHC class I and II as well as the immunogenicity (Calis *et al.* 2013). This results in a tab-delimited list of predicted neoantigens.

### 2.2 Benchmarking using available datasets

ScanNeo2 was assessed using the TESLA (Wells *et al.* 2020) dataset that contains data obtained from subjects (*n* = 8) in metastatic melanoma and non-small cell lung cancer. In that study, the authors tested 608 peptides for immunogenicity, of which 37 were found to be immunogenic. We analyzed the data using ScanNeo2 (see Supplementary Data for details), which included the data preparation (Supplementary Table S2) and identified in each patient 9152–96 819 putative HLA-binding peptides that account for unique peptides (Supplementary Table S3). Among those, 5671–34 981 candidate neoantigens originate from a single source, such as alternative splicing, exitron-splicing, gene fusion, indels, or SNVs. ScanNeo2 detects 35 of the 37 experimentally validated immunogenic peptides but also captures 476 non-immunogenic peptides. In the following, we applied ScanNeo2 with more stringent parameters (immunogenicity score ≥0.5, TPM ≥2, ranking score ≥1000, and VAF ≥0.02), which reduces the number of non-immunogenic peptides to 391 while retaining 34 of the validated peptides (Supplementary Table S4). Similar settings in nextNEOp detects less immunogenic peptides. In addition, we looked at the runtime and memory requirements of ScanNeo2. In comparison to nextNEOpi, ScanNeo2 requires on average ∼22.5% more CPU time. The main reason for that is the extensive variant calling in which multiple sources are used with high sensitivity.

## 3 Conclusion

We introduce ScanNeo2, a comprehensive snakemake-based pipeline for predicting tumor neoepitopes from sequencing data. It has been implemented in snakemake to ensure ease of installation, usage as well as high portability. In contrast to other workflows, ScanNeo2 incorporates multiple sources, thereby providing a means to decipher the neoantigen landscape to an unprecedented degree.

## Supplementary Material

btad659_Supplementary_Data

## Data Availability

WES and RNA-seq data used in this article are available in the Synapse platform, at https://www.synapse.org/#!Synapse:syn21048999.
