# Supplementary material for: ScanNeo2: a comprehensive workflow for neoantigen detection and immunogenicity prediction from diverse genomic and transcriptomic alterations
Source: Bioinformatics. 2023 Oct 26;39(11):btad659. doi: 10.1093/bioinformatics/btad659 (PMC10629934; doi:10.1093/bioinformatics/btad659)
Supplement: btad659_Supplementary_Data [file btad659_supplementary_data.pdf]

SUPPLEMENTARY INFORMATION

ScanNeo2: a comprehensive workflow for neoantigen detection and immunogenicity prediction from diverse genomic and transcriptomic alterations

Richard. A. Schäfer,[1] Qingxiang Guo[1] and Rendong Yang[1,2*]

[1]Department of Urology, Northwestern University Feinberg School of Medicine, 303 E Superior St, 60611 Chicago, Illinois, USA and [2]Robert H. Lurie Comprehensive Cancer Center, Northwestern University Feinberg School of Medicine, 303 E Superior St, Chicago, IL 60611, USA

*Corresponding author. rendong.yang@northwestern.edu

Feature comparison of existing workflows

Table S1. Feature comparison of common workflows for the prediction of neoantigens.

method	sources	input	prepro-cessing	mhc class	hla typing	immuno-genicity	batch processing	publication
`ScanNeo2`	SNVs, indels, exitrons, gene fusions, canonical splicing	WES/WGS and/or RNA-seq, as raw FASTQ or BAM, or pre-computed variants in VCF	yes	I,II	yes	yes	yes	this study
`INTEGRATE-neo`	gene fusions	gene model as GenePred and gene fusions as BEDPE	no	I	yes	no	no	Zhang *et al.* (2017)
`nextNEOpi`	SNVs, indels, gene fusions	WES/WGS and RNA-seq or WES/WGS only, as raw FASTQ files	yes	I,II	yes	yes	yes	Rieder *et al.* (2022)
`pVACtools`	gene fusions, variants	pre-computed variants in VCF (annotated with VEP) annotated fusions (from AGFusion, Arriba)	yes	I,II	no	no	no	Hundal *et al.* (2020)
`ScanNeo`	indels, exitrons	RNA-seq as BAM	no	I	yes	no	no	Wang *et al.* (2019)
`TSNAD v2.0`	SNVs, indels	WES/WGS as raw FASTQ	yes	I	yes	no	no	Zhou *et al.* (2017)

Methods

Pre-processing & Alignment

ScanNeo2 is a comprehensive snakemake-based pipeline to predict tumor neoantigens from raw or pre-processed DNA sequencing data, such as whole-exome or whole-genome sequencing (WES/WGS), and/or RNA sequencing data. It is implemented in the snakemake (Mölder *et al.*, 2021) workflow language to enable maximum reproducibility. The installation process is simplified using conda environments and Docker containers, which are automatically fetched, installed, and executed by snakemake, thereby eliminating the need for users to manually install tools and dependencies. Fig. A1 depicts the different rules in the workflow. To run the pipeline, users can provide tumor/normal FASTQ or BAM files of either WES/WGS and/or RNA-seq data. In addition, pre-computed variants in VCF are also supported. When the sequencing reads are provided in FASTQ format, an optional pre-processing step can be invoked. Here, quality control of the reads is performed before and after the pre-processing using FastQC v0.12.1 (www.bioinformatics.babraham.ac.uk/projects/fastqc/). For the actual pre-processing procedure, fastp v0.23.4 (Chen *et al.*, 2018) is used, which incorporates automated removal of adapter contamination. If the pre-processing is omitted, FASTQC is only applied to the input data. In the following, the RNA-seq reads are aligned to the reference genome (hg38) using STAR v2.7.10b. Here, the read groups are determined using either the sample identifiers or are directly extracted from BAM files (when provided). In other words, the former indicates that reads of different read groups need to be specified in separate files. In the following, the aligned reads are post-processed and the duplicates are removed using samtools v1.16.1 (Li *et al.*, 2009; Danecek *et al.*, 2021). If ScanNeo2 has been configured to perform indel/SNV calling, the reads are subsequently realigned using BWA v0.7.17 (Li and Durbin, 2009). In that regard, the WES/WGS data are solely aligned using BWA.

HLA genotyping

In the HLA genotyping, WES/WGS and/or RNA-seq reads are used to determine the HLA alleles. In the case of RNA-seq data, the STAR alignments are used. For HLA class I alleles, the data is filtered HLA reads using yara (Siragusa *et al.*, 2013), and subsequently the aligned reads are subjected to Optitype v1.3.5. In a similar manner, HLA-HD (Kawaguchi *et al.*, 2017) is used to determine HLA class II alleles.

Variant Calling

ScanNeo2 integrates different modules for the variant calling. These include alternative splicing, exitron-splicing, and gene fusion events that are solely detected on RNA-seq data. As a consequence, these methods are omitted in the absence of RNA-seq data. In contrast, the SNV/indel detection is done either on WES/WGS, RNA-seq, or both.

Alternative Splicing

Alternative splicing events are identified using SplAdder v3.0.4 (Kahles *et al.*, 2016). We considered all event types (e.g., exon skips, intron retentions, alternative 3' splice sites, alternative 5' splice sites, mutually exclusive exons, multiple (coordinated) exons skips). As SplAdder only reports the events in *Hierarchical Data Format* (HDF5) and TXT format, ScanNeo2 converts the event files into VCF. ScanNeo2 mainly applies the default parameters of spladder, but confidence level (option confidence), and the number of iterations (option iterations) can be specified in the configuration. The former controls how strongly input alignments are filtered before new nodes and edges are added to the splicing graph. Here, the default is set to the highest level of confidence (default: 3, the maximum level of filtering). The latter controls the number of iterations when adding new intron edges (default: 5).

Exitron Splicing

We used ScanExitron (Wang *et al.*, 2021) to detect exitron-derived splicing events. Here, the mapping quality is used that is specified for the whole analysis. In addition, default values for AO and PSO cutoffs are used. Subsequently, the results were converted into VCF using the provided scripts.

Gene Fusion

In this case, Arriba (Uhrig *et al.*, 2021) is used. ScanNeo2 applies the recommended settings for the STAR alignments to use in Arriba. In addition, parameters such as the minimum number of supported reads, the maximum E-value can be specified, whereas others are left to the default values. The resulting fusion events in tab-delimited format are converted into VCF using the provided scripts.

SNVs/Indels

ScanNeo2 employs multiple tools which include mutect2 and haplotypecaller from GATK (Van der Auwera and O'Connor, 2020) for SNVs and short indels, and transIndel (Yang *et al.*, 2018) for long indels. However, ScanNeo2 allows to call these individually. It is to be noted, that this is applied both on the WES/WGS and RNA-seq data. Here, the (re-)alignments from BWA are used. Scanneo2 follows the best practice approach for short variant discovery. At first, the alignments are subjected to haplotypecaller which calls germline variants and is used with a recent build of the dbSNP database (Sherry *et al.*, 2001). Subsequently, the variants are recalibrated using the *Variant Quality Score Recalibration* (VQSR) method that first builds a model using known, highly validated variant resources. This selects a subset of variants within the callset of high significance that functions as training set. Overlaps of the training/truth resources sets and the detected callsets are used to model the distribution of these variants and groups them into clusters and assign so-called VQSLOD scores to all variants. In the next step, the filtering thresholds are applied to the variants in which the authors recommend a truth sensitivity level of 99.5% and 99% for SNV and indels, respectively. This results in filtered variants call sets for SNVs, and indels which are used for the base recalibration. The recalibrated alignment files

are then applied to haplotypecaller, and the variants are again recalibrated and filtered to end up with call sets for SNVs and indels. Similarly, the recalibrated BAM file is subjected to mutect2 which identifies somatic variants. Subsequently, the indels are filtered and the SNVs and indels are sorted into separate files. Finally, the transIndel is used on the data to detect long indels. This is followed by the removal of PCR slippage events.

Annotation and Priorization

In the following, the individual VCF files are annotated using the Variant Effect Predictor (McLaren *et al.*, 2016). Here, we used the plugin for nonsense-mediate decay events and predict the corresponding peptide sequences. For that, the flanking regions of the corresponding mutant alleles are extracted to the corresponding lengths that match the length of the corresponding peptides. This also allows to consider frameshift deletions that escape NMD. By default, ScanNeo2 determines peptide sequences of 8-11 for class I pMHCs, and 13-25 for class II pMHCs. Subsequently, netMHCpan and netHMCII (Reynisson *et al.*, 2020) are used with the genotyped HLA alleles and the peptides. Peptides that fall short of a binding affinity 500nm are discarded. The surviving peptides are subjected to the immunogenicity caluclation for which the prediction tools from IEDB were used. Also the transcript expression was assessed. In addition, ScanNeo2 proves the ranking score to determine the significance for the detected pMHC. This is defined as $score = \frac{1}{B} + F + A * 100$ in which B corresponds to the binding affinity, F as the fold-change between WT and MT alleles and A as the variant allele frequency.

Benchmarking

For the benchmarking of ScanNeo2, the TESLA datasets (Wells *et al.*, 2020) was used (https://www.synapse.org/#!Synapse:syn21048999). In particular, normal/tumor whole exome sequencing (WES), and tumor RNA-Seq data of six patients in melanoma and two patients in non-small-cell lung carcinoma (NSCLC) was used. The samples were subjected to pre-processing applying a minimum length of 10, and an average quality score of 20. In addition, the window trimming with a window size of 3 and quality of 20 has been applied. In the following, stringent settings were used in the the variant calling. The results are listed in Table S3. Other parameters were set to default values. In a more stringent settings, the TPM was set to reads > 2, ranking score > 1000, and variant allele frequency was set to 0.02 and the immunogenicity score was set to 0.5. Table S4 summarizes the findings.

Runtime and Memory Requirements

All samples were subjected to each module individually. We tested single patient data in which the runtime between nextNEOpi took about 1.83min per million reads, wherease ScanNeo2 required 2.245min. However, this increase can be mainly attributed to the somatic variant calling that accounts for 0.7min.

Table S2. Statistics of the pre-processing and alignments when analysing the TESLA dataset using ScanNeo2. Percentages in brackets refer to the raw sequencing reads.

dataset	patient	gender	tumor type	pathological status	data type	raw reads	pre-processed	aligned	post-processed	realigned
TESLA_1	#1	male	melanoma	metastatic	WES	77,315,047	76,452,210 (98.9%)	76,415,779 (98.8%)	46,960,350 (60.7%)	-
TESLA_2	#1	male	melanoma	-	WES	65,065,156	64,399,484 (99%)	64,372,058 (98.9%)	39,660,762 (61%)	-
TESLA_3	#1	male	melanoma	metastatic	RNA-seq	114,418,684	113,764,124 (99.4%)	111,696,268 (97.6%)	71,757,006 (62.7%)	71,683,102 (62.6%)
TESLA_9	#2	male	melanoma	metastatic	WES	92,134,713	90,743,303 (98.5%)	90,709,341 (98.5%)	52,905,576 (57.4%)	-
TESLA_10	#2	male	melanoma	-	WES	84,393,221	83,212,965 (98.6%)	83,174,058 (98.6%)	48,305,616 (57.2%)	-
TESLA_11	#2	male	melanoma	metastatic	RNA-seq	128,200,209	127,608,888 (99.5%)	122,712,220 (95.7%)	68,320,301 (53.3%)	68,234,627 (53.2%)
TESLA_17	#3	male	melanoma	metastatic	WES	93,903,632	93,169,589 (99.2%)	93,125,671 (99.2%)	54,495,429 (58%)	-
TESLA_18	#3	male	melanoma	-	WES	91,810,687	91,004,222 (99.1%)	90,959,851 (99.7%)	50,512,775 (55%)	-
TESLA_19	#3	male	melanoma	metastatic	RNA-seq	210,304,828	209,381,632 (99.6%)	200,540,903 (95.4%)	101,200,451 (48.1%)	100,989,265 (48%)
patient_04_tumor	#4	female	melanoma	primary	WES	206,408,268	203,592,333 (98.6%)	102,074,320 (49.5%)	100,898,773 (48.9%)	-
patient_04_normal	#4	female	melanoma	-	WES	204,364,392	201,112,330 (98.4%)	100,583,786 (49.2%)	99,148,139 (48.5%)	-
patient_04_tumor_rna	#4	female	melanoma	primary	RNA-seq	52,817,666	52,501,151 (99.4%)	52,298,030 (99%)	40,233,677 (76.1%)	40,212,295 (76.1%)
patient_08_tumor	#8	female	melanoma	primary	WES	95,205,908	95,027,214 (99.8%)	47,602,818 (50%)	42,217,724 (44.3%)	
patient_08_normal	#8	female	melanoma	-	WES	107,863,636	107,623,837 (99.8%)	53,927,283 (50.0%)	47,237,924 (43.8%)	-
patient_08_tumor_rna	#8	female	melanoma	primary	RNA-seq	64,925,125	64,628,244 (99.5%)	62,415,917 (96.1%)	31,535,196 (48.6%)	31,502,315 (48.5%)
patient_09_tumor	#9	unknown	melanoma	primary	WES	115,096,082	114,798,555 (99.7%)	57,547,899 (50%)	47,017,529 (40.9%)	-
patient_09_normal	#9	unknown	melanoma	-	WES	117,295,606	117,077,071 (99.8%)	58,645,421 (50.0%)	46,634,050 (39.8%)	-
patient_09_tumor_rna	#9	unknown	melanoma	primary	RNA-seq	74,516,294	74,258,161 (99.7%)	71,619,658 (96.1%)	40,233,550 (54%)	40,209,673 (54%)
TESLA_87	#12	male	NSCLC	primary	WES	32,396,607	31,412,436 (97%)	31,405,200 (96.9%)	23,338,801 (72%)	-
TESLA_86	#12	male	NSCLC	-	WES	34,026,801	32,803,864 (96.4%)	32,796,858 (96.4%)	25,783,551 (75.8%)	-
TESLA_94	#12	male	NSCLC	primary	RNA-seq	91,331,492	90,563,764 (99.2%)	89,092,658 (97.5%)	35,223,956 (38.6%)	35,126,975 (38.5%)
TESLA_91	#16	female	NSCLC	primary	WES	31,255,039	29,588,688 (94.7%)	29,582,344 (94.6%)	23,338,801 (74.7%)	-
TESLA_90	#16	female	NSCLC	-	WES	42,288,812	36,293,815 (85.8%)	36,285,915 (85.8%)	28,203,757 (66.7%)	-
TESLA_97	#16	female	NSCLC	primary	RNA-seq	84,591,682	83,654,909 (98.9%)	82,411,134 (97.4%)	30,971,215 (36.6%)	30,834,319 (36.5%)
total						2,311,929,587	2,284,672,789 (98.8%)	1,841,995,390 (79.7%)	1,196,134,909 (51.7%)	

Table S3. ScanNeo2 results on the TELSA dataset

patient	total pMHC	unique peptides	single source	TESLA pMHC	TESLA imm. pMHC	TESLA non-imm.
#1	93616	11959	7651	46	7	39
#2	153310	29988	17212	49	5	44
#3	174110	96819	34981	76	12	63
#4	49312	10935	7512	25	2	23
#8	185171	37261	17809	66	1	65
#9	186412	34657	25456	55	2	53
#12	51512	9303	6761	53	3	50
#16	33656	9152	5671	101	3	98

Table S4. ScanNeo2 results on the TELSA dataset with more stringent parameters

patient	total pMHC	unique peptides	single source	TESLA pMHC	TESLA imm. pMHC	TESLA non-imm.
#1	6778	3677	578	26	7	33
#2	15123	11766	3516	47	5	43
#3	24145	17661	7881	68	11	58
#4	5781	3677	2231	21	2	23
#8	27811	19871	6771	56	1	44
#9	31212	25455	9817	45	2	48
#12	19122	5667	1231	48	3	47
#16	12912	6781	2312	80	3	95

Supplementary Figures

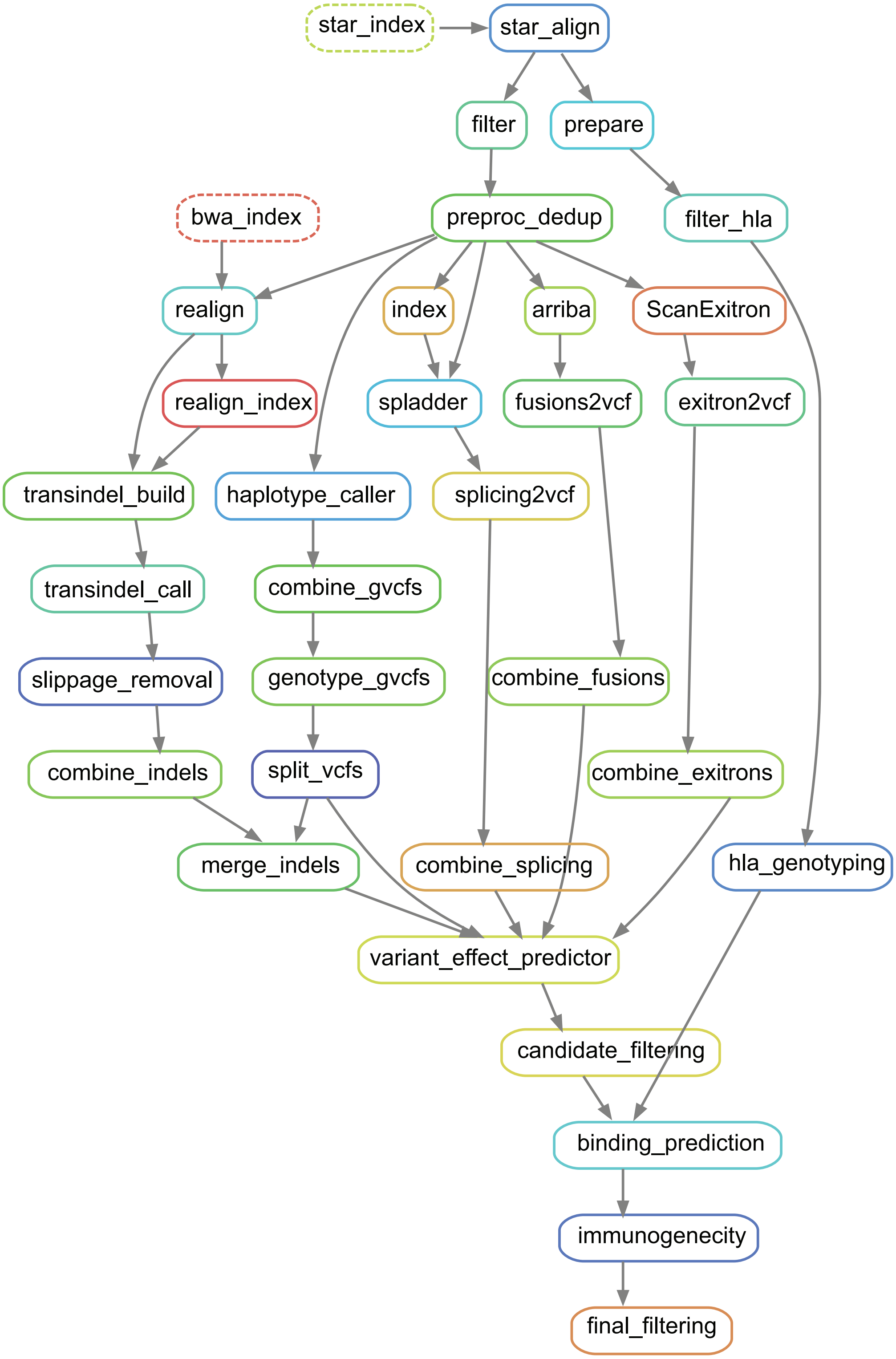

Fig. A1. Visualization of ScanNeo2 workflow and the interplay of the rules as *directed acyclic graph*

References

Chen, S. *et al.* (2018). fastp: an ultra-fast all-in-one FASTQ preprocessor. *Bioinformatics*, **34**(17), i884–i890.

Danecek, P. *et al.* (2021). Twelve years of SAMtools and BCFtools. *GigaScience*, **10**(2), giab008.

Hundal, J. *et al.* (2020). pVACtools: A Computational Toolkit to Identify and Visualize Cancer Neoantigens. *Cancer Immunol. Res.*, **8**(3), 409–420.

Kahles, A. *et al.* (2016). SplAdder: identification, quantification and testing of alternative splicing events from RNA-Seq data. *Bioinformatics*, **32**(12), 1840–1847.

Kawaguchi, S. *et al.* (2017). HLA-HD: An accurate HLA typing algorithm for next-generation sequencing data. *Hum. Mutat.*, **38**(7), 788–797.

Li, H. and Durbin, R. (2009). Fast and accurate short read alignment with Burrows–Wheeler transform. *Bioinformatics*, **25**(14), 1754–1760.

Li, H. *et al.* (2009). The Sequence Alignment/Map format and SAMtools. *Bioinformatics*, **25**(16), 2078–2079.

McLaren, W. *et al.* (2016). The Ensembl Variant Effect Predictor. *Genome Biol.*, **17**(1), 1–14.

Mölder, F. *et al.* (2021). Sustainable data analysis with Snakemake. *F1000Research*, **10**(33), 33.

Reynisson, B. *et al.* (2020). NetMHCpan-4.1 and NetMHCIIpan-4.0: improved predictions of MHC antigen presentation by concurrent motif deconvolution and integration of MS MHC eluted ligand data. *Nucleic Acids Res.*, **48**(W1), W449–W454.

Rieder, D. *et al.* (2022). nextNEOpi: a comprehensive pipeline for computational neoantigen prediction. *Bioinformatics*, **38**(4), 1131–1132.

Sherry, S. T. *et al.* (2001). dbSNP: the NCBI database of genetic variation. *Nucleic Acids Res.*, **29**(1), 308–311.

Siragusa, E. *et al.* (2013). Fast and accurate read mapping with approximate seeds and multiple backtracking. *Nucleic Acids Res.*, **41**(7), e78.

Uhrig, S. *et al.* (2021). Accurate and efficient detection of gene fusions from RNA sequencing data. *Genome Res.*, **31**(3), 448–460.

Van der Auwera, G. A. and O'Connor, B. D. (2020). *Genomics in the Cloud*. O'Reilly Media, Inc., Sebastopol, CA, USA.

Wang, T.-Y. *et al.* (2019). ScanNeo: identifying indel-derived neoantigens using RNA-Seq data. *Bioinformatics*, **35**(20), 4159–4161.

Wang, T.-Y. *et al.* (2021). A pan-cancer transcriptome analysis of exitron splicing identifies novel cancer driver genes and neoepitopes. *Mol. Cell*, **81**(10), 2246–2260.e12.

Wells, D. K. *et al.* (2020). Key Parameters of Tumor Epitope Immunogenicity Revealed Through a Consortium Approach Improve Neoantigen Prediction. *Cell*, **183**(3), 818–834.e13.

Yang, R. *et al.* (2018). Indel detection from DNA and RNA sequencing data with transIndel. *BMC Genomics*, **19**(1), 1–11.

Zhang, J. *et al.* (2017). INTEGRATE-neo: a pipeline for personalized gene fusion neoantigen discovery. *Bioinformatics*, **33**(4), 555–557.

Zhou, Z. *et al.* (2017). TSNAD: an integrated software for cancer somatic mutation and tumour-specific neoantigen detection. *R. Soc. Open Sci.*, **4**(4), 170050.
